# Does long-term creatine supplementation impair kidney function in resistance-trained individuals consuming a high-protein diet?

**DOI:** 10.1186/1550-2783-10-26

**Published:** 2013-05-16

**Authors:** Rebeca Lugaresi, Marco Leme, Vítor de Salles Painelli, Igor Hisashi Murai, Hamilton Roschel, Marcelo Tatit Sapienza, Antonio Herbert Lancha Junior, Bruno Gualano

**Affiliations:** 1 School of Physical Education and Sport - Laboratory of Applied Nutrition and Metabolism, University of Sao Paulo, Av Mello de Moraes, 65, Sao Paulo, SP 05508-030, Brazil; 2 School of Medicine – Division of Rheumatology, University of Sao Paulo, Av Mello de Moraes, 65, Sao Paulo, SP 05508-030, Brazil; 3 School of Medicine – Division of Nuclear Medicine, University of Sao Paulo, Av Mello de Moraes, 65, Sao Paulo, SP 05508-030, Brazil; 4 School of Physical Education and Sport - Laboratory of Neuromuscular Adaptations to Strength Training, University of Sao Paulo, Av Mello de Moraes, 65, Sao Paulo, SP 05508-030, Brazil

**Keywords:** Glomerular filtration rate, ^51^Cr-EDTA clearance, Side effects

## Abstract

**Background:**

The aim of this study was to determine the effects of creatine supplementation on kidney function in resistance-trained individuals ingesting a high-protein diet.

**Methods:**

A randomized, double-blind, placebo-controlled trial was performed. The participants were randomly allocated to receive either creatine (20 g/d for 5 d followed by 5 g/d throughout the trial) or placebo for 12 weeks. All of the participants were engaged in resistance training and consumed a high-protein diet (i.e., ≥ 1.2 g/Kg/d). Subjects were assessed at baseline (Pre) and after 12 weeks (Post). Glomerular filtration rate was measured by ^51^Cr-EDTA clearance. Additionally, blood samples and a 24-h urine collection were obtained for other kidney function assessments.

**Results:**

No significant differences were observed for ^51^Cr-EDTA clearance throughout the trial (Creatine: Pre 101.42 ± 13.11, Post 108.78 ± 14.41 mL/min/1.73m^2^; Placebo: Pre 103.29 ± 17.64, Post 106.68 ± 16.05 mL/min/1.73m^2^; group x time interaction: F = 0.21, p = 0.64). Creatinine clearance, serum and urinary urea, electrolytes, proteinuria, and albuminuria remained virtually unchanged.

**Conclusions:**

A 12-week creatine supplementation protocol did not affect kidney function in resistance-trained healthy individuals consuming a high-protein diet; thus reinforcing the safety of this dietary supplement.

**Trial registration:**

ClinicalTrials.gov NCT01817673

## Background

Creatine supplementation has been recognized as one of the most efficient dietary supplements capable of increasing muscle strength and lean mass [1], as well as high-intensity exercise performance [2]. However, the indiscriminate use of this supplement has raised concerns regarding its safety, especially in relation to kidney function [3].

Despite the increasing number of publications showing that creatine supplementation may not affect kidney function in humans [4-10], it has been recommended that the chronic effects of creatine supplementation should be better examined in some specific populations [3]. In this regard, there is an empirical claim that creatine supplementation might pose a risk at those consuming protein in excess. In fact, there is compelling evidence indicating that high-protein diets can accelerate renal deterioration in individuals with chronic kidney disease [11], although it is unknown whether this holds true in healthy persons [12].

Resistance-trained practitioners often consume a high-protein diet along with creatine supplements in an attempt to enhance power/strength and lean mass. The alleged “kidney overload” caused by creatine (and its by-product creatinine) and excessive protein ingestion merits further investigation. Therefore, the purpose of this study was to examine the effects of creatine supplementation on kidney function in resistance-trained individuals consuming a high-protein diet. In most of the previous human studies involving creatine supplementation, kidney function was assessed via serum creatinine or its derivative equations. However, the spontaneous conversion of creatine into creatinine [13] may falsely suggest decreased kidney function in creatine-supplemented individuals [8]. To overcome this potential drawback, we used a gold standard method - ^51^Chromium-ethylenediamine tetraacetic acid (^51^Cr-EDTA) clearance - to accurately measure glomerular filtration rate in this study.

## Methods

### Subjects

Young healthy males who regularly engaged in resistance training for at least 1 year and were ingesting a high-protein diet (≥ 1.2 g/Kg/d; which is a usual prescription to resistance-trained practitioners [14]) were eligible to participate. The exclusion criteria included: vegetarian diet, use of creatine supplements in the past 6 months, chronic kidney disease, and use of anabolic steroids. The participants were advised to maintain their habitual diet. Participants’ characteristics are presented in Table 1. The study was approved by the Ethical Advisory Committee from the School of Physical Education and Sport, University of Sao Paulo. All of the participants signed the informed consent. This trial was registered at clinicaltrials.gov as NCT01817673.

**Table 1:** Participants’ characteristics

	**Creatine (n = 12)**	**Placebo (n = 14)**
Age (years)	24 (3)	27 (5)
Height (m)	1.79 (0.08)	1.78 (0.05)
Weight (Kg)	80.4 (10.3)	78.4 (12.4)
BMI (Kg/m^2^)	24.8 (1.6)	24.7 (2.9)
Training experience (years)	5 (2)	7 (3)
Training frequency (sessions per week)	5 (1)	4 (1)

Data expressed as mean (standard deviation).

### Experimental protocol

A 12-week, double-blind, randomized, placebo-controlled trial was conducted between July 2011 and February 2013 in Sao Paulo, Brazil. The participants were randomly assigned to receive either creatine or placebo in a double-blind fashion. All of the participants continued with their usual resistance training routines throughout the study. The participants were assessed at baseline (Pre) and after 12 weeks (Post). ^51^Cr-EDTA clearance was performed to measure the glomerular filtration rate. Additionally, blood samples and twenty-four-hour urine collection were obtained following a 12-h overnight fasting for kidney function assessments. Dietary intake was assessed by 7-day food diaries. The participants were asked to refrain from strenuous physical activity 24 h prior to the pre- and posttests. Food intake was assessed by 7-day food diaries. This method consists of the listing of foods and beverages consumed during 7 consecutive days. Energy and macronutrients were analyzed by the Dietpro® 5i software (Sao Paulo, Brazil).

### Creatine supplementation protocol and blinding procedure

The creatine group received creatine monohydrate (20 g/d for 5 d followed by 5 g/d throughout the trial). The placebo group received the same dosage of dextrose. The participants were advised to consume their supplements preferably along with meals (*e.g.,* breakfast, lunch, afternoon snack, and dinner). The supplement packages were coded so that neither the investigators nor the participants were aware of the contents until the completion of the analyses. In order to verify the purity of the creatine used, a sample was analyzed by high-performance liquid chromatography (HPLC). This established 99.9% of purity, with no other peaks detected (creatinine, dicyandiamide, and cyclocreatine < 0.01%).

### ^51^Cr-EDTA clearance

After a 24h-protein-restricted diet and a 12-h overnight fasting, the participants were admitted to the clinical research center at 7:00 a.m., where they rested in a supine position with an indwelling polyethylene catheter inserted into a cubital vein in both arms. A single dose of 3.7 MBq (100 μCi) of the ^51^Cr-EDTA tracer, in a volume of 1 ml was injected intravenously in the right arm. The catheter was flushed through with 10 ml of saline. Accurately timed 10-ml blood-samples were drawn into a heparinized tube from the opposite arm at 4 and 6 h after the injection. The plasma disappearance curve was designed using the results of these time-points. To measure the radioisotope activity, the blood samples were centrifuged at 1500 *g* for 10 min and 3 ml of plasma was measured in a well-calibrated counter (Genesys Genii™, LabLogic Systems Inc, Brandon, Florida, USA) for the energy of chromium-51 (320 keV). Each sample, including 3 ml of standard solution taken as an aliquot from 3.7 MBq (100 μCi) ^51^Cr-EDTA diluted to 500 mL in saline, was counted for 5 min. The plasma clearance rate was calculated by the slope-intercept method with a single-compartment model, which assumes that the tracer spreads out immediately after injection in its volume of distribution. The Brochner–Mortensen method was used for correcting systematic errors of the slope-intercept technique according to the following equation:

$${\mathrm{Cl}}_{c}=0.990{8\;\times \;\mathrm{Cl}}_{\mathrm{nc}}{-0.001218\;\times \;\mathrm{Cl}}_{\mathrm{nc}}{}^{2}$$

where Cl_c_ is the clearance corrected for the first exponential and Cl_nc_ is the non-corrected clearance. Systematic errors caused by an abnormal radioisotope distribution were corrected using the Groth method. ^51^Cr-EDTA clearance was also corrected for 1.73 m^2^ body surface area. The coefficient of variation (CV) for ^51^Cr-EDTA clearance was 9.7%.

### Blood and urinary analyses

Blood samples were obtained from an antecubital vein, following a 12-h overnight fasting. Participants followed their normal diet consumption during the 24-h urine collection. Urine samples were stored at approximately 4°C. Both blood and urinary measurements were performed in the morning. Creatinine was determined using Jaffe’s kinetic method. Urinary and serum sodium and potassium were assessed by using a flame photometer (FP8800, Kruss®, Hamburg, Germany). Urea was assessed by an UV-kinetic method. Albuminuria was determined by nephelometry and proteinuria was measured through the benzethonium chloride method.

All of the samples were analyzed in duplicate and the CV were 2.0, 2.2, 1.1, 2.1, 2.3, 5.3, 24.5, and 16.4% for serum creatinine, serum sodium, serum potassium, serum urea, proteinuria, albuminuria, urinary sodium, and urinary potassium, respectively.

### Statistical analysis

It was determined that 24 participants is necessary to provide 80% power (5% significance, two-tailed) to detect a 20% reduction in the ^51^Cr-EDTA clearance. In order to account for mid-trial withdrawals, we enlarged our study sample size to 46 participants.

Data were tested by a Mixed Model with Kenward-Roger adjustment for unbalanced group sizes, using the software SAS 9.2 (SAS Institute Inc., Cary, NC, USA). Group (creatine and placebo) and time (Pre and Post) were considered as fixed factors and participants were defined as a random factor. A post hoc test adjusted by Tukey was planned to be used whenever a significant F-value was detected. The between-group difference in the ratio of participants who had reduction in the ^51^Cr-EDTA clearance was tested by the Chi-square (*χ*2) test. Significance level was previously set at p < 0.05. Data are presented as mean and standard deviation.

## Results

### Flux of participants

**Figure 1:**
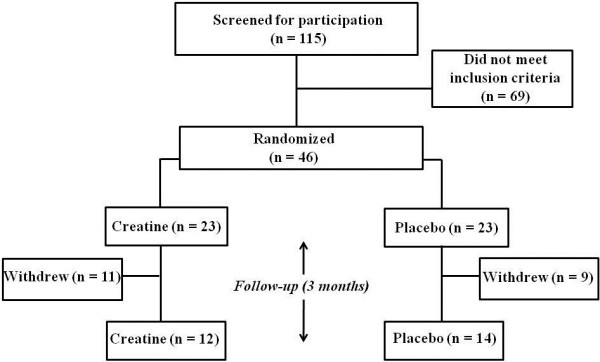
Fluxogram of participants.

The flux of participants is shown in Figure 1. A total of 115 volunteers who were screened for participation and 69 volunteers did not meet the inclusion criteria. The remaining 46 participants were randomly assigned to either the creatine (n = 23) or the placebo (n = 23) group. Afterwards, 15 participants withdrew for personal reasons (8 from the creatine group and 7 from the placebo group). Additionally, 5 participants (3 from the creatine group and 2 from the placebo group) did not attend the post-intervention assessment; hence, they were removed from the analysis. Therefore, 12 participants in the creatine group and 14 participants in the placebo group were analyzed (n = 26).

### Food intake

Table 2 shows the food intake data. Protein intake ranged from 1.2 to 3.1 g/Kg/d. Diet remained unchanged throughout the study.

**Table 2:** Food intake before (Pre) and after 12 weeks (Post) of either creatine or placebo supplementation in resistance-trained individuals consuming a high-protein diet

	**Creatine (n = 12)**		**Placebo (n = 14)**		
***Variable***	***Pre***	***Post***	***Pre***	***Post***	***P (group x time interaction)***
Protein (g)	154 (45)	154 (39)	133 (36)	120 (39)	0.54
Carbohydrate (g)	283 (70)	322 (96)	271 (92)	272 (124)	0.49
Lipid (g)	84 (23)	91 (27)	98 (31)	86 (31)	0.23
Protein (%)	25 (5)	23 (5)	22 (4)	22 (5)	0.65
Carbohydrate (%)	45 (6)	47 (9)	43 (10)	47 (6)	0.58
Lipid (%)	30 (6)	30 (8)	35 (8)	32 (6)	0.48
Total Energy (Kcal)	2506 (530)	2725 (522)	2518 (544)	2368 (781)	0.29
Protein/ body weight (g/Kg)	1.9 (0.5)	1.9 (0.5)	1.7 (0.5)	1.6 (0.5)	0.53

Data expressed as mean (standard deviation). There were no significant differences between groups at baseline. No significant within- or between-group differences were noted.

### Kidney function assessments

**Figure 2:**
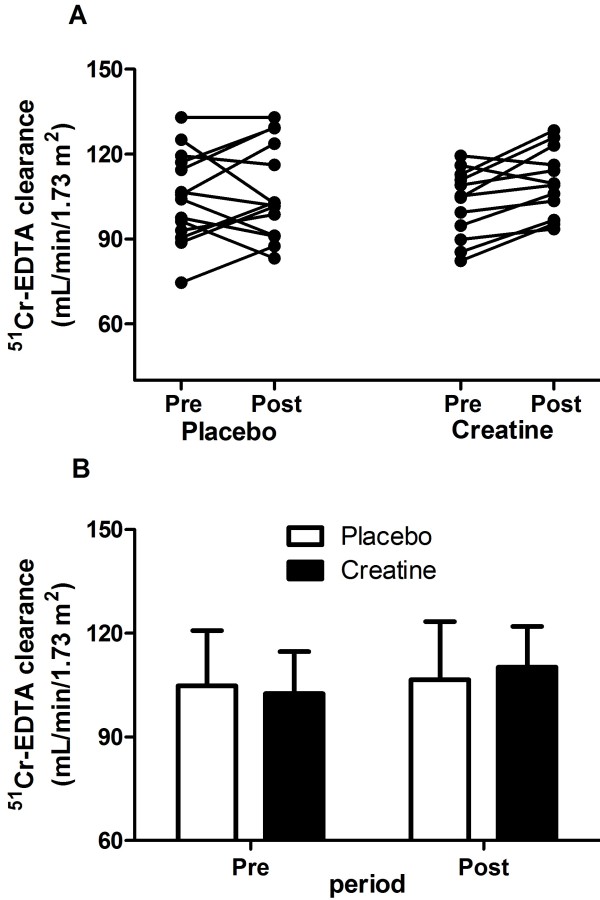
^**51**^**Cr-EDTA clearance before (Pre) and after 12 weeks (Post) of either creatine (n = 12) or placebo (n = 14) supplementation in resistance-trained individuals consuming a high-protein diet.** Panel **A**: individual data. Panel **B**: mean ± standard deviation. No significant difference between groups across time (group x time interaction) was observed (F = 0.21, p = 0.64). *Note:* Conversion factors for units: glomerular filtration rate in mL/min/1.73 m^2^ to mL/s/1.73 m^2^, ×0.01667.

Figure 2 shows the data regarding the ^51^Cr-EDTA clearance. There were no significant differences between groups at Pre or Post (group × time interaction: F = 0.21, p = 0.64). In the creatine group, 2 out of 12 participants had a decrease in the ^51^Cr-EDTA clearance, whereas 6 out of 14 participants experienced reduction in the ^51^Cr-EDTA clearance in the placebo group (P(*χ*^2^ > 2.081) = 0.149).

Table 3 presents the data regarding albuminuria, proteinuria, serum and urinary sodium and potassium, serum urea and serum creatinine. There were no significant differences between groups for any of the parameters (p > 0.05). None of the participants had either albuminuria or proteinuria.

**Table 3:** Kidney function parameters before (Pre) and after 12 weeks (Post) of either creatine or placebo supplementation in resistance-trained individuals consuming a high-protein diet

	**Creatine (n = 12)**		**Placebo (n = 14)**		
***Variable***	***Pre***	***Post***	***Pre***	***Post***	***P (group x time interaction)***
Albuminuria (mg/24 h)	19 (38)	15 (28)	8 (7)	4 (2)	0.99
Proteinuria (g/24 h)	0.14 (0.11)	0.14 (0.10)	0.10 (0.05)	0.10 (0.07)	0.83
Urinary potassium (mEq/24 h)	65 (24)	59 (22)	68 (24)	65 (19)	0.86
Urinary sodium (mEq/24 h)	231 (56)	226 (91)	195 (65)	191 (52)	0.99
Serum potassium (mEq/L)	4 (0.3)	4 (0.4)	5 (0.4)	4 (0.4)	0.26
Serum sodium (mEq/L)	141 (3)	141 (2)	142 (3)	141 (4)	0.53
Serum creatinine (mg/dL)	1.1 (0.1)	1.2 (0.2)	1.0 (0.1)	1.1 (0.1)	0.30
Serum urea (mg/dL)	41.7 (10.7)	39.2 (11.7)	33.3 (6.7)	33.4 (7.2)	0.63

Data expressed as mean (standard deviation). There were no significant differences between groups at baseline. No significant within- or between-group differences were noted. *Note:* Conversion factors for units: serum creatinine in mg/dL to mol/L, ×88.4; serum urea in mg/dL to mmol/L, ×0.166; glomerular filtration rate in mL/min/1.73 m^2^ to mL/s/1.73 m^2^, ×0.01667.

## Discussion

The present results are in agreement with other investigations that have demonstrated the safety of creatine supplementation on kidney function in distinct populations [4-9]. However, most of these studies estimated glomerular filtration rate by using serum endogenous markers, which are widely used in clinical practice but potentially susceptible to methodological errors. For instance, serum creatinine and its derivative equations are influenced by dietary intake, particularly by creatine-containing foods or supplements. Upon the ingestion of creatine, one may expect an increase in serum creatinine, since creatine is spontaneously and irreversibly converted into creatinine. As such, a false positive diagnosis of a decreased kidney function may occur in creatine-supplemented individual when only serum creatinine data are taken into consideration. Although serum creatinine was not significantly elevated in the current study, previous observations from our group [8] and others [15] support the inaccuracy of creatinine-based markers in the evaluation of kidney function in creatine-supplemented individuals. To circumvent this potential bias, we measured glomerular filtration rate using the gold-standard technique ^51^Cr-EDTA clearance, which allowed us to properly conclude that creatine supplementation did not affect kidney function in this study.

Applying the above mentioned technique, we previously showed that 35 days of creatine supplementation did not alter kidney function in a 20-year-old man with a single kidney [16]. Moreover, we reported that 3 months of creatine supplementation had no deleterious effect on kidney function in post-menopausal women [9] and in type-2 diabetic patients [17], corroborating the safety of this supplement. The present data extend this notion to typical creatine consumers, suggesting that healthy resistance-trained individuals can “deal” with creatine supplementation even in combination with a higher level of protein intake (considering the Recommended Dietary Intake (RDI) of 0.8 g/Kg/d). In consonance with our findings, a few cross-sectional studies have shown no significant differences in kidney function between higher and lower protein consumers [18,19]. In fact, given the human habituation to the high-nitrogenous diet throughout the span of evolution, these findings might not be considered unexpected. Yet, further prospective studies must explore the impact of chronic nitrogenous-rich diets upon kidney function in healthy individuals.

This study is not without limitations. First, the follow-up of this study is too short, precluding any definitive conclusions. Originally, this trial was designed to cover a 12-month period. However, a drastic withdrawal rate forced us to reduce the follow-up period. Therefore, trials of longer treatment duration are warranted. Second, we selected recreationally trained participants to increase the ecological validity of this study, since this population is thought to be the largest consumer of creatine supplements. However, it is possible that highly-trained athletes taking anabolic steroids and under exhaustive resistance training regimens may experience a differential response to creatine supplementation. Finally, it is worth noting that all of the individuals were apparently healthy, so that these data cannot be extrapolated to individuals with, or at risk of, chronic kidney diseases. In such conditions, creatine users must be systematically monitored for kidney function.

## Conclusions

Three months of creatine supplementation did not have a detrimental effect on kidney function in resistance-trained practitioners consuming a high-protein diet (i.e., ≥ 1.2 g/Kg/d).

## Abbreviations

Cr-EDTA: ^51^Chromium-ethylenediamine tetraacetic acid; Pre: baseline; Post: after 12 weeks; CV: coefficient of variation; BMI: body mass index; RDI: Recommended Dietary Intake; χ2: Chi-square.

## Competing interests

The authors declare that they have no conflict of interest.

## Authors’ contributions

RL and BG were significant manuscript writers; ML, HR, MTS, and AHLJ were significant manuscript revisers/reviewers; BG, HR, and AHLJ participated in the concept and design; RL, ML, VSP, and MTS were responsible for data acquisition; BG, HR, VSP, and RL participated in data analysis and interpretation. All authors read and approved the final manuscript.
